# A Simple Cautery

**Published:** 1877-06

**Authors:** T. C. Stellwager

**Affiliations:** American Journal of Med. Science.


### ARTICLE IX.

### *A Simple Cautery.*

One of the most useful means of applying the actual cautery has been apparently neglected or allowed to pass unheeded up to the present time, the simplicity of the method being probably the cause of its escaping attention.

It has long been a desirable thing, with the practitioner of dentistry, to be able to accomplish the cauterization of what is termed sensitive dentine, often found where the dental caries has attacked the necks of the teeth, near the margins of the gums; most of the cauterants in actual use being unreliable, or to a degree unmanageable, and liable to penetrate deeper into the structure, or injure the mucous membrane by running over it. This, to a certain degree, has been avoided by the use of the galvanic cautery; but the apparatus required for this purpose is both costly and cumbersome, beside being easily deranged, and somewhat difficult to apply to certain surfaces where, by the undercutting of the caries, the platinum point requires to be bent or hooked.

While operating on the 13th of March, for my friend, Dr. J. S. Walker, I attempted, by the use of a minute coal of fire upon a match-stick, to obtund the sensation of the superficial portion of such a cavity, as above described; meeting with some difficulty in the breaking off of the heated portion, he suggested the use of a harder wood, and I immediately ignited the end of a stick of dental pivot wood, which wood, from its characteristics, being both dry and compressed, proved a most satisfactory and inexpensive means of obtaining the desired effect. It has since appeared to me that sticks of hickory, or any combustible substance that is dense, tough, and not readily consumed in the ordinary atmosphere, might be of service to the general surgeon, but particularly where the throat, nares, ear, uterus, or anus, are the points to be cauterized; or for the physician, where immediate vesication is demanded, it could be conveniently used. These sticks might be made more inflammable by soaking in something like a solution of saltpetre, before drying and passing through the process of condensation, which dentists accomplish by an ordinary draw-plate, such as is used for making wire.

To use this, a suitable portion should be burned in the flame of an ordinary match for a few moments, and then, by blowing out the flame, the incandescent portion at the point may be brought to the shape desired, and the temperature raised by passing rapidly through the air, or, *vice versa*, lowered by allowing a trifling coating of ash to accumulate upon the surface. This will burn thus for one or more minutes, according as more or less is charred by the flame, and one or more of the small sticks are used singly or tied together, or the stick made of larger diameter.

It might also be that a tube of some non-conducting material might be filled with an ordinary lampwick, previously prepared, and by a spring regulated to keep the ignited portion of the combustible material constantly pressed out at one end.—*Prof. T. C. Stellwager, in American Journal of Med. Science.*

